# Modulation of the microhomology-mediated end joining pathway suppresses large deletions and enhances homology-directed repair following CRISPR-Cas9-induced DNA breaks

**DOI:** 10.1186/s12915-024-01896-z

**Published:** 2024-04-29

**Authors:** Baolei Yuan, Chongwei Bi, Yeteng Tian, Jincheng Wang, Yiqing Jin, Khaled Alsayegh, Muhammad Tehseen, Gang Yi, Xuan Zhou, Yanjiao Shao, Fernanda Vargas Romero, Wolfgang Fischle, Juan Carlos Izpisua Belmonte, Samir Hamdan, Yanyi Huang, Mo Li

**Affiliations:** 1 Bioscience Program, Biological and Environmental Science and Engineering Division, King Abdullah University of Science and Technology (KAUST), Thuwal, 23955-6900 Kingdom of Saudi Arabia; 2 Beijing Advanced Innovation Center for Genomics (ICG), Biomedical Pioneering Innovation Center (BIOPIC), School of Life Sciences, College of Chemistry, College of Engineering, Peking-Tsinghua Center for Life Sciences, Peking University, Beijing, China; 3 Institute for Cell Analysis, Shenzhen Bay Laboratory, Shenzhen, China; 4 Bioengineering Program, Biological and Environmental Science and Engineering Division, King Abdullah University of Science and Technology (KAUST), Thuwal, 23955-6900 Kingdom of Saudi Arabia; 5 Altos Labs, Inc, San Diego, CA 92121 USA; 6 Present address: King Abdullah International Medical Research Center (KAIMRC), King Saud bin Abdulaziz University for Health Sciences (KSAU-HS), Ministry of National Guard Health Affairs (MNG-HA), Jeddah, Saudi Arabia

**Keywords:** CRISPR-Cas9 genome editing, Large deletions, Microhomology-mediated end joining, Homology-directed repair

## Abstract

**Background:**

CRISPR-Cas9 genome editing often induces unintended, large genomic rearrangements, posing potential safety risks. However, there are no methods for mitigating these risks.

**Results:**

Using long-read individual-molecule sequencing (IDMseq), we found the microhomology-mediated end joining (MMEJ) DNA repair pathway plays a predominant role in Cas9-induced large deletions (LDs). We targeted MMEJ-associated genes genetically and/or pharmacologically and analyzed Cas9-induced LDs at multiple gene loci using flow cytometry and long-read sequencing. Reducing *POLQ* levels or activity significantly decreases LDs, while depleting or overexpressing *RPA* increases or reduces LD frequency, respectively. Interestingly, small-molecule inhibition of *POLQ* and delivery of recombinant RPA proteins also dramatically promote homology-directed repair (HDR) at multiple disease-relevant gene loci in human pluripotent stem cells and hematopoietic progenitor cells.

**Conclusions:**

Our findings reveal the contrasting roles of *RPA* and *POLQ* in Cas9-induced LD and HDR, suggesting new strategies for safer and more precise genome editing.

**Supplementary Information:**

The online version contains supplementary material available at 10.1186/s12915-024-01896-z.

## Background

CRISPR-Cas9 can introduce double strand breaks (DSBs) to a specific genomic locus that shares sequence complementarity with the CRISPR guide RNA (gRNA). The DSBs can be repaired through different cellular mechanisms, including the classical non-homologous end joining (C-NHEJ, hereafter referred to as NHEJ), MMEJ (also called alternative NHEJ), homologous recombination (HR), and single-stranded annealing (SSA) pathways. NHEJ often generates small insertions and deletions (indels) [1] and is believed to be the dominant repair pathway for DSBs induced by CRISPR-Cas9 [2]. MMEJ relies on small homologies for DNA repair, while SSA requires longer ones. HR is an error-free DNA repair mechanism that requires a homologous template.

The majority of on-target modifications induced by CRISPR-Cas9 were believed to be indels less than 20 bp in length according to numerous large-scale studies on Cas9 cleavage outcomes [1, 3–5]. However, more recent work [6–10] revealed frequent on-target large deletions (LDs) and large complex rearrangements of the genome caused by CRISPR-Cas9. One of the reasons that LDs or complex genomic rearrangements eluded detection in earlier studies is that they analyzed genome editing outcomes with Sanger and/or short-read next-generation sequencing of short PCR amplicons (usually < 300 bp), which are unable to resolve large genomic alterations. Long-read sequencing platforms, such as PacBio and Oxford Nanopore, which are much better at resolving large rearrangements, have been used for the analysis of genome editing outcomes [6, 7, 11–13].

The LD issue can have significant implications for the application of the otherwise versatile genome editing tool CRISPR-Cas9. A previous study has investigated 32 potential candidates from different DNA repair pathways that might affect LDs in mouse ESCs and found promising genes in both NHEJ and MMEJ repair pathways [14]. However, the underlying mechanism of LD is not fully understood and strategies to reduce LDs are urgently needed. A previous study showed a high occurrence of microhomology (MH) at Cas9-induced LDs [15], suggesting potential involvement of MMEJ repair pathway. Our investigation aligns with this observation, revealing a significant enrichment of MHs at the breakpoint junctions of LDs induced by CRISPR-Cas9. Hence, we hypothesized that MMEJ plays roles in generating LDs following CRISPR-Cas9 cleavage and investigated the roles of four key MMEJ genes—poly [ADP-ribose] polymerase 1 (PARP1), replication protein A (RPA), DNA polymerase theta (POLQ), and DNA ligase 3 (LIG3)—in LD formation. In brief, PARP1 binds to the ends of DSBs as a competitor of Ku that is known to play a similar role in the NHEJ pathway [16] and activates the end resection [17], thus channeling DNA repair to the MMEJ pathway. RPA that contains three subunits—RPA1, RPA2, and RPA3—binds resected single-stranded DNA (ssDNA) and triggers homologous recombination (HR) [18, 19]. RPA also prevents ssDNA annealing thus further blocking MMEJ repair [20]. POLQ plays multiple roles in MMEJ, including promoting DNA synapse formation and ssDNA annealing, extending overhangs [21], and inhibiting HR [22]; therefore, POLQ is considered to play a central role in MMEJ in higher organisms [23]. LIG3 is a major ligase for sealing the gaps in the last step of MMEJ [23, 24].

We found that depletion or inhibition of POLQ or overexpression of RPA significantly reduced LD frequency. By contrast, knocking down PARP1 or LIG3 had no effect on LD frequency. We further provided base-resolution validation of the observations by using a long-read individual molecule sequencing method, IDMseq [7]. We also found small-molecule inhibition of POLQ or delivery of recombinant RPA proteins significantly increased HDR efficiency. These results highlight the role of MMEJ in CRISPR-Cas9-mediated genome editing and provide potential targets and strategies for safe and precise genome editing.

## Results

### Most CRISPR-Cas9-induced LDs in human pluripotent stem cells contain microhomology at breakpoint junctions

**Fig. 1:**
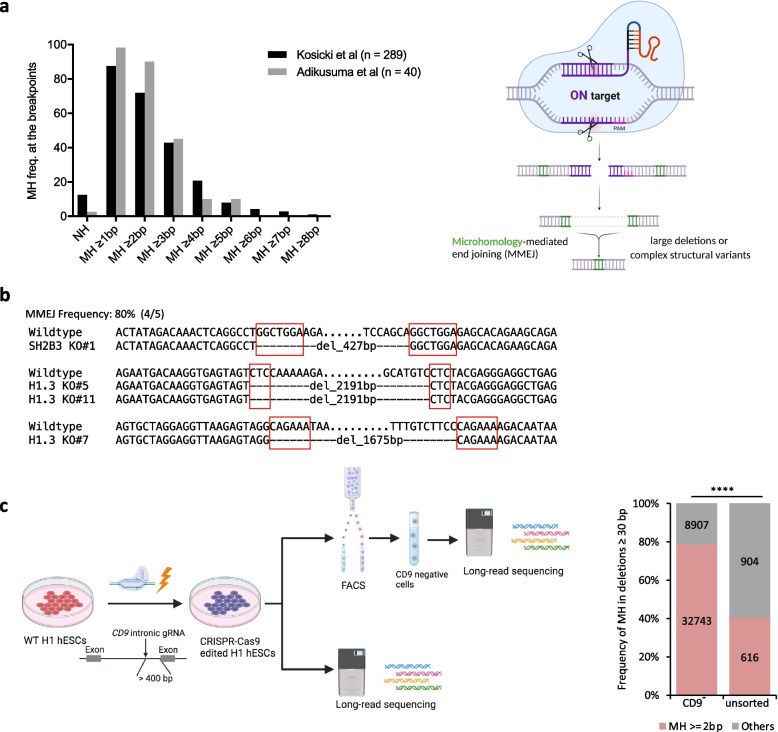
MH is enriched at the breakpoint junctions of CRISPR-Cas9-induced LDs. **a** Left: analysis of microhomology (MH) frequency at Cas9-induced breakpoint junctions in two published data [6, 8]; right: schematic of how MMEJ could lead to CRISPR-induced LDs (created with BioRender.com). NH: no homology. **b** LD events detected in Cas9-edited human pluripotent cell lines. Red color boxes indicate MH sequences. **c** Left: schematic of the strategy to analyze CRISPR-induced LDs in the CD9 locus (created with BioRender.com); right: MH frequency in deletions ≥ 30 bp quantified from long-read sequencing data. ****p < 0.0001, Fisher’s exact test

CRISPR-Cas9 can efficiently cut target DNA to promote gene knockout through the formation of small indels or precise installation of DNA sequence changes through homology directed repair (HDR). However, it also causes unintended LDs and structural variations (SVs) of up to several megabases or even whole chromosome loss [6–8, 25, 26]. The underlying mechanism of CRISPR-Cas9-induced LD remains unclear. We collated sequencing data of 329 CRISPR-Cas9-edited alleles from two published studies [6, 8] and found an unusually high frequency of MHs at LD breakpoint junctions. For example, MHs ≥ 2 bp were present in more than 70% of the LD alleles (Fig. 1a). We also randomly examined 20 clones of human pluripotent stem cell (hPSC) lines edited by CRISPR-Cas9 in the *SH2B3* and *H1.3* genes in-house and found five of them harbored LD alleles, in which four contained MHs (Fig. 1b; Additional file 1: Fig. S1a).

To provide quantitative evidence for prevalent MHs in Cas9-edited loci, we edited the *CD9* and *PIGA* intronic regions, respectively, in H1 human embryonic stem cells (hESCs) using Cas9/gRNA ribonucleoprotein (RNP) complex (Fig. 1c; Additional file 1: Fig. S1b). At both loci, the distance between the intronic gRNA and the nearest exons is more than 200 bp. Therefore, the edited cells that lose cell surface expression of *CD9* or *PIGA*, as monitored by fluorescence-activated cell sorting (FACS), are considered to contain LDs that extend at least from the CRISPR-Cas9 cleavage site to the nearest exon. We performed PacBio circular consensus sequencing of a 7-kb region flanking the intronic gRNA target amplified from the CD9-negative or both PIGA-positive and PIGA-negative cells and respective unsorted cells (Fig. 1c; Additional file 1: Fig. S1b). The sequencing data showed that most PIGA-negative sorted cells contain LDs in which the nearby exon was deleted either entirely or partially, while the PIGA-positive sorted cells often contain small indels and occasionally LDs that the nearby exon was not disrupted (Additional file 1: Fig. S1c), which indicates that the FACS-based quantification can be used for LD studies. The examination of reads with deletions ≥ 30 bp at the Cas9 cut site revealed a strong enrichment of MHs (≥ 2 bp) in the breakpoint junctions in the negative populations (78.61% and 97.91% in the *CD9* and *PIGA* loci, respectively), which was lessened in the unsorted populations (Fig. 1c; Additional file 1: Fig. S1d). Since MMEJ-mediated DNA repair results in MHs at the breakpoint junctions (Fig. 1a), the high occurrence of MHs suggests that the MMEJ pathway plays roles in meditating CRISPR-induced LDs.

### RPA and POLQ regulate LD formation, but not PARP1 and LIG3

**Fig. 2:**
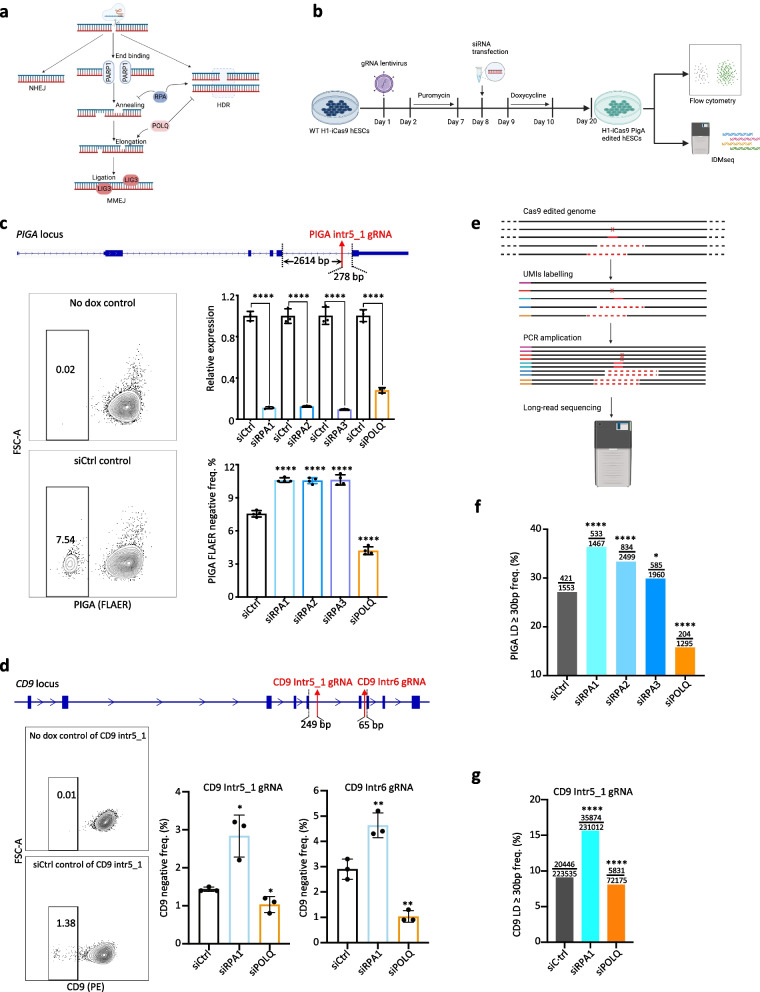
*RPA* and *POLQ* play opposite roles in CRISPR-Cas9-induced LDs. **a** Schematic of the roles of four key genes in the MMEJ pathway (created with BioRender.com). **b** Diagram of the workflow for the knockdown experiments (created with BioRender.com). **c** Top: the location of the *PIGA* intronic gRNA, the numbers indicate the distances between gRNA cut site and the adjacent exons; bottom left: example flow cytometry analysis of *PIGA* expression using the FLAER assay; bottom right: normalized mRNA level of siRNA target genes biological replicates *n* = 3, and LD frequency quantified by FACS, biological replicates *n* = 4, ****p < 0.0001. **d** Top: the location of the *CD9* intronic gRNAs, the number indicates the distance between the gRNA cut site, and the nearest exon; bottom left: example flow cytometry analysis of *CD9* expression using the PE anti-CD9; bottom right: LD frequency quantified by FACS, biological replicates *n* = 3, *p < 0.05, **p < 0.01. **e** Schematic of IDMseq analysis of LDs (created with BioRender.com). **f** Frequencies of LDs (≥ 30 bp) quantified by IDMseq. The numerator indicates the LD event number, and the denominator indicates the total event number detected by IDMseq. ****p < 0.0001, *P < 0.05, Fisher’s exact test. **g** Frequencies of LDs (≥ 30 bp) quantified by ONT long-read sequencing. The numerator indicates the LD read number, and the denominator indicates the total read number. ****p < 0.0001, Fisher’s exact test

To better understand the role of the MMEJ pathway in the formation of LDs, we modulated the function of four genes (*PARP1, RPA, POLQ*, and *LIG3*) in hPSCs undergoing CRISPR-Cas9 editing (Fig. 2a, b). To achieve consistent and uniform induction of CRISPR-Cas9 editing, we used an H1 ESC line with a doxycycline-inducible Cas9 expression system integrated into the AAVS1 safe harbor locus (H1-iCas9) [27]. We firstly quantified the frequency of LDs using sgRNAs targeting different intronic regions of the X-linked *PIGA* and autosomal *CD9* genes, which are established models [3, 6, 14] for the study of CRISPR-Cas9 editing outcomes (sgRNA positions are shown in Additional file 1: Fig. S1e and S3a). Thirteen intronic sgRNAs targeting *PIGA* and 7 intronic sgRNAs targeting *CD9* were individually expressed in H1-iCas9 ESCs using a constitutive lentiviral vector. Upon doxycycline (dox) induction, these sgRNAs guided Cas9 to generate DSBs located 65–2441 bp from the nearest exon (Additional file 1: Fig. S1e and S3a). Subsequent DNA repair could lead to small indels that did not reach coding sequences and preserved gene expression or to LDs that extended into nearby exons and disrupted gene expression, which resulted in cells being stained positively and negatively in flow cytometry, respectively (Additional file 1: Fig. S1c). Control sgRNAs targeting the exons resulted in an almost 100% PIGA knockout, as indicated by the FLAER FACS assay (Additional file 1: Fig. S1e). This outcome suggested that our system achieved a saturating level of editing efficiency.

The intronic sgRNA data showed that the frequency of PIGA-deficient cells (FLAER^neg^) ranged from 0.23 to 9.05% (average 3.52 ± 0.83%) in Cas9-edited cells (Additional file 1: Fig. S1e). In the case of the autosomal *CD9* gene, intronic sgRNAs led to lower frequencies of CD9-negative cells, ranging from 0.19 to 4.4% (average 1.25% ± 1.44%) (Additional file 1: Fig. S3a). Note that the negatively stained population is likely a conservative estimate of LD because LDs extending to the opposite side of the nearest exon may not result in loss of expression (i.e., FLAER^neg^) (Additional file 1: Fig. S1c) and in-frame LDs may lead to hypomorphic levels (i.e., cells with intermediate FLAER staining in Additional file 1: Fig. S1e). In cells without Cas9 expression (no dox), background LD events were almost undetectable (Fig. 2c, d and Additional file 1: S2a). This observation demonstrates that the LDs were specifically caused by Cas9-induced DSBs. LD frequency could not be predicted based solely on the orientation of the sgRNA (targeting the + or − strand) or the distance between the sgRNA and the nearest exon (Additional file 1: Fig. S1e, f), suggesting a dependency on the sequence context. CRISPR/Cas9-mediated genome editing has been associated with potential induction of various severe chromosome structural abnormalities, such as chromosome loss [26, 28], truncation [29–31], and translocation [32–34]. To rule out the effect of such events on the LD%, we edited the H1-iCas9 ESCs employing intr5_1 sgRNA, the highest LD% intronic gRNA based on our test (Additional file 1: Fig. S1e), and quantified the X chromosome copy number using a well-established qPCR-based assay [35] by targeting multiple gene loci (*VCX*, *PNPLA4*, *TSPAN7*, *USP9X*, *USP27X*, and *HRRT1*) flanking *PIGA* gene in both non-edited cells and PIGA FLAER-negative sorted cells. We did not find significant chromosome loss at these loci in PIGA FLAER negative sorted cells (Additional file 1: Fig. S1g). To corroborate this result, we also conducted a ddPCR assay for the *WAS* gene (located on the same X chromosome p-arm as the *PIGA* gene), which is considered a more sensitive quantification method for detecting chromosome copy number variation. Consistently, the ddPCR result did not show any significant difference in X chromosome copy number. Thus, we established an optimal and sensitive setup to evaluate the effects of modulating the MMEJ pathway on the occurrence of LDs in the rest of our study.

Considering the prevalence of MHs in LDs, we hypothesized that LD frequency could be controlled by modulating the activity of the MMEJ pathway. We knocked down four key MMEJ pathway genes, *PARP1*, *LIG3*, *RPA* (including *RPA1*, *RPA2*, and *RPA3*), and *POLQ* in H1-iCas9 cells expressing the *PIGA* intr5_1 sgRNA (Fig. 2a–c, Additional file 1: S2a-c) and induced Cas9 expression 24 h later. LD frequency was monitored by FACS analysis of FLAER staining as described in the preceding paragraph (Fig. 2b, c, and Additional file 1: S2a). The results showed that knocking down POLQ caused a 40% reduction in LD frequency while knocking down RPA proteins led to a 40% increase in LD frequency (Fig. 2c). Since the knockdown of PARP1 or LIG3 consistently showed little effect on LD frequency (Additional file 1: Fig. S2c), they were excluded from further investigation.

To confirm the impact of POLQ and RPA on LDs at other gene loci, we conducted knockdown experiments targeting the *CD9* gene. Consistent FACS results were observed across experiments using intr5_1, intr6, and intr7 gRNAs. Knockdown of POLQ significantly reduced the frequency of CD9^neg^ cells (by up to 65%) whereas knockdown of RPA1 led to a twofold increase in CD9^neg^ frequency (Fig. 2d; Additional file 1: S3b).

To investigate whether the LD frequency is influenced by the cell state, we examined the impact of the cell cycle on LD prevalence. Human PSCs are notoriously difficult to arrest in the G1 phase. Among all the cell cycle synchronization drugs and protocols we tested, only nocodazole at 100 ng/ml could synchronize hPSCs reliably without toxicity (Additional file 1: Fig. S2d, e), which is consistent with a previous publication [36]. The H1-iCas9/PIGA intr5_1 sgRNA system we used is highly sensitive to doxycycline exposure, leading to the maximum LD frequency within a 12-h doxycycline treatment (Additional file 1: Fig. S2f). We observed a slight increase in LD frequency when cells were arrested at the G1 phase, while no significant differences were found across all cell cycle phases (Additional file 1: Fig. S2g). We further checked the effects of the knockdown of MMEJ protein on the cell cycle. The cell cycle profile of the knockdown samples did not differ significantly from that of the control (Additional file 1: Fig. S2h). Therefore, our findings suggest that the LD frequency is mostly contributed by DNA repair pathway inhibition.

To better quantify the Cas9 editing outcomes in MMEJ-knockdown H1-iCas9 cells at base resolution, we next performed IDMseq [7] of the *PIGA* locus. Briefly, individual genomic regions flanking the Cas9 cut site were labeled with a unique molecular identifier (UMI) and amplified for long-read PacBio sequencing (Fig. 2e). In the following sequencing data analysis, we referred to deletions ≥ 30 bp as LDs. Consistent with previous studies, IDMseq showed that the vast majority of SVs detected in Cas9-edited cells were LDs [7]. The baseline LD frequency of the control siRNA as per IDMseq was higher than that estimated by FACS, which is expected because FLAER^neg^% underestimates LD frequency as discussed above and because LDs of 30–278 bp in size (i.e., noncoding deletions) are only detectable by IDMseq (Additional file 1: Fig. S1b, c). We observed that the LD length spectrum exhibits striking similarities across all groups (Additional file 1: Fig. S2k), implying that the LD size remains unaffected by the MMEJ deficiency. Consistent with the FACS analysis, the IDMseq results also confirmed that POLQ knockdown decreased LD frequency and RPA knockdown increased it (Fig. 2f). Consistent observations were made at the *CD9* locus using Oxford Nanopore Technologies (ONT) long-read sequencing to quantify LD frequency (Fig. 2g). To gain insights into the impact of POLQ and RPA knockdown on DSB repair, we conducted MH analysis for LD events of *PIGA*. Our results did not yield statistically significant differences when comparing the MMEJ knockdown groups with the control group, possibly due to the constraints imposed by the sequencing depth of long-read data. However, a discernible trend emerged in the data, indicating an increase in MH ≥ 2bp in RPA-knockdown cells and a corresponding decrease in POLQ-knockdown cells (Additional file 1: Fig. S2l). This trend aligns with the observed patterns in LD data (Fig. 2f).

### LD induced by CRISPR-Cas9 can be controlled by modulating POLQ and RPA

**Fig. 3:**
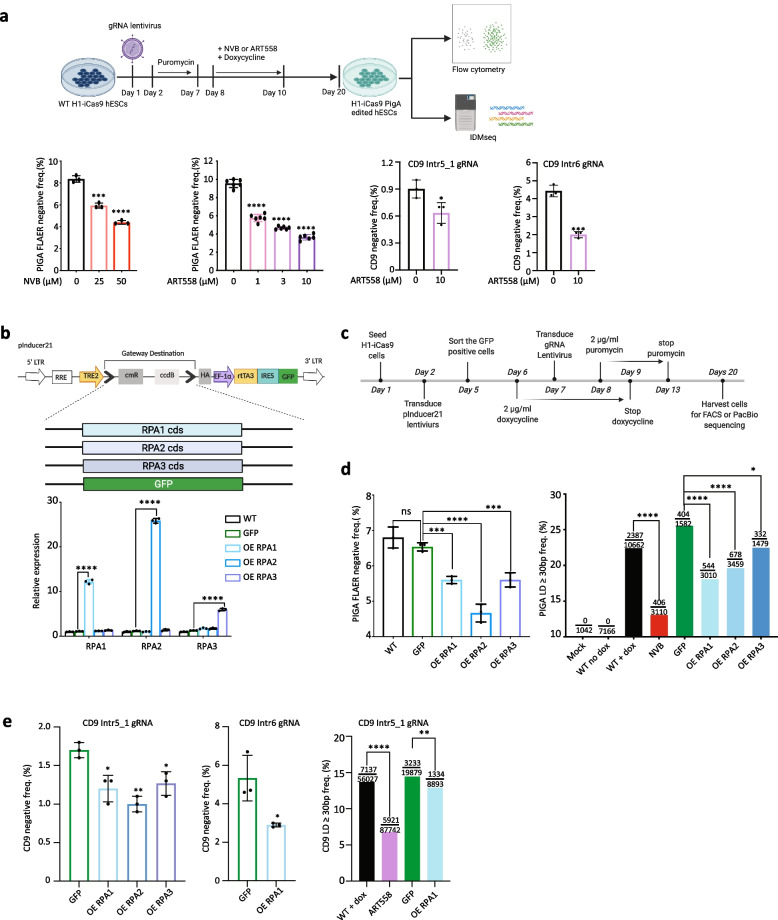
CRISPR-Cas9-induced LDs can be suppressed by inhibiting POLQ or overexpressing RPA. **a** Top: schematic of the workflow for the POLQ inhibition experiment (created with BioRender.com); bottom: LD frequency quantified by FACS, biological replicates *n* = 3, *p < 0.05, ***p < 0.001, ****p < 0.0001. **b** Top: schematics of the inducible RPA constructs; bottom: the mRNA level of RPA genes after doxycycline treatment for two days. **c** The workflow of RPA overexpression experiments. **d, e** Left**:** LD frequency quantified by FACS, biological replicates *n* = 3, *p < 0.05, **p < 0.01, ***p < 0.001, ****p < 0.0001, ns: not significant, two-sided Student’s *t*-test; OE: overexpression. Right: frequency of LD (≥ 30 bp) quantified by IDMseq and ONT sequencing. The numerator indicates the LD event number, and the denominator indicates the total event number detected by IDMseq. *p < 0.05, **p < 0.01, ****p < 0.0001, Fisher's exact test

POLQ is an error-prone polymerase and is upregulated in numerous cancers [37–41]. The antibiotic novobiocin (NVB) has recently been identified as a specific inhibitor of POLQ. NVB inhibits the ATPase activity of POLQ through direct binding to its ATPase domain and thus phenocopies POLQ depletion and impairs MMEJ DNA repair in human cells [39]. We therefore used NVB to test whether targeting the specific MMEJ-related activity of POLQ could recapitulate the reduction of LDs mediated by POLQ knockdown. NVB was introduced to the cells during the induction of Cas9 expression by doxycycline in the *PIGA* intr5_1 sgRNA-positive H1-iCas9 ESCs. NVB decreased the frequency of LDs by up to 50% in a dose-dependent manner (Fig. 3a). NVB showed no discernible effect on the pluripotency of treated hESCs (Additional file 1: Fig. S2i). High concentrations of NVB (50 µM) showed signs of cytotoxicity, while lower concentrations were well tolerated by the cells (Additional file 1: Fig. S2j). We also performed the LD analysis on both the *PIGA* and *CD9* loci using a potent and selective inhibitor of the polymerase function of POLQ–ART558 [42]. Similarly, ART558 treatment significantly decreased the frequency of LDs by up to 61.78% in a dose-dependent manner (Fig. 3a; Additional file 1: Fig. S3c). Therefore, transient inhibition of POLQ function is sufficient to reduce the formation of LDs following repair of DSBs induced by Cas9.

The RPA proteins prevent ssDNA annealing, thus blocking MMEJ repair [20, 43]. Consistently, we showed that knocking down RPA increases LD frequency. We hypothesized that increasing RPA availability could divert DNA repair away from the MMEJ pathway during Cas9 editing and lead to a reduction of LDs. Therefore, we cloned three RPA subunits (RPA1, RPA2, and RPA3) and GFP (as a control) individually into an inducible lentiviral expression vector, pInducer21, that expresses GFP constitutively (Fig. 3b). Successfully transduced cells were sorted based on GFP positivity and transgene expression was induced by doxycycline. The expression level of the transgenic RPA proteins increased from sixfold to 26-fold after doxycycline induction without affecting the expression of other RPA subunits (Fig. 3b). Such levels of overexpression of RPA proteins resulted in significant reductions in LD frequency at both *PIGA* and *CD9* loci, as detected by FACS (Fig. 3c–e; Additional file 1: Fig. S3c).

To gain a sequence-level understanding of the effect of POLQ inhibition and RPA overexpression on the DNA repair outcome of CRISPR-Cas9 editing, we performed IDMseq [7] of the *PIGA* locus and ONT long-read sequencing of the *CD9* locus as in the knockdown experiments. Similar to the knockdown results, no substantial disparity in the LD size spectrum was discernible across the samples (Additional file 1: Fig. S2m). Both POLQ inhibition and RPA overexpression demonstrated the ability to decrease CRISPR-Cas9-induced LDs (Fig. 3d, e), consistent with the FACS analysis results. Interestingly, our MH analysis unveiled an unexpected reduction in the GFP control group, with the underlying mechanism remaining elusive. The MH ≥ 2bp frequency exhibited a decrease when comparing the NVB treatment and RPA overexpression groups to the wild-type group (Additional file 1: Fig. S2n).

To investigate the generality of the effects of POLQ and RPA on LDs, we conducted additional experiments on the X-linked gene *LAMP2* in an induced pluripotent stem cell (iPSC) line [44] and quantified the LD using flow cytometry. Knocking down RPA subunits significantly increased LD frequency, while inhibiting POLQ with NVB and ART558 or overexpressing RPA subunits significantly reduced it (Additional file 1: Fig. S3d-g). Additionally, we performed bulk ONT long-read sequencing to quantify LD in two disease-associated CRISPR-edited genes (*WAS* and *HBB*) in the same cellular models used for *PIGA* and *CD9* editing. Consistent with other gene loci, LD frequency significantly increased in the RPA-knockdown group and dramatically decreased in POLQ deficiency or RPA overexpression groups (Additional file 1: Fig. S3h, i).

To examine whether modulating POLQ activity or RPA overexpression affects the desirable small indel formation, we analyzed the editing efficiency of a sgRNA targeting PIGA exon 2 by FACS (Additional file 1: Fig. S2o). The data showed that treatment with NVB or overexpression of RPA1, RPA2, or RPA3 did not change the frequency of PIGA knockout cells (the majority of which contain small indels). These results showed that LD can be controlled by inhibiting POLQ activity and overexpressing RPA without changing the overall editing efficiency. Together, the data suggested that small-molecule inhibition of POLQ and RPA overexpression could offer convenient and safe ways to reduce unwanted LDs following Cas9 editing without compromising editing efficiency.

### Modulating POLQ and RPA improves HDR efficiency

**Fig. 4:**
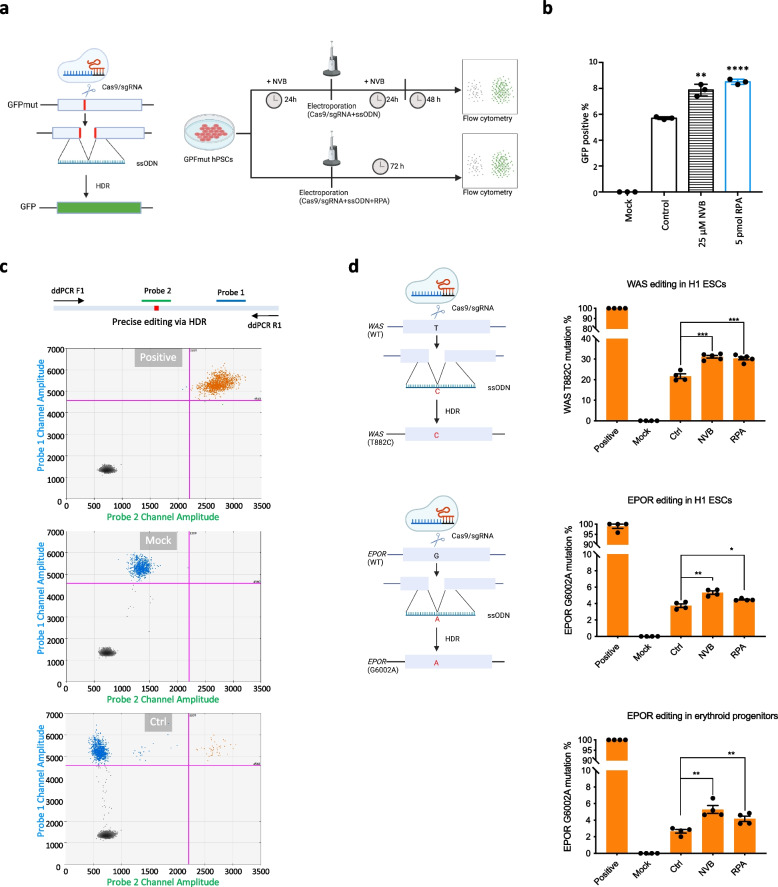
Modulation of POLQ and RPA increases HDR efficiency. **a** Schematic of mutant GFP correction by CRISPR-mediated HDR (left) and strategies to improve HDR efficiency (right) (created with BioRender.com). The green color indicates the restoration of green fluorescence. **b** The frequency of GFP positive cells quantified by FACS, biological replicates *n* = 3, **p < 0.01, ****p < 0.0001. **c** Schematic of ddPCR probe-based assay design for detecting CRISPR-mediated precise mutation via HDR and representative 2D plots of ddPCR events from positive (an EPOR G6002 mutant cell line), mock (non-edited line), and control (edited in *EPOR* locus) H1 ESC samples (data were shown in Fig. 4d). Probe 2 is designed to specifically recognize the installed point mutation but not wild-type sequence. Probe 1 is designed to recognize both mutant and wild-type sequences. **d** Schematic of CRISPR-mediated HDR via editing *WAS* and *EPOR* genes (left) (created with BioRender.com), and the HDR frequency analyzed by ddPCR (right), biological replicates *n* = 4, *p < 0.01, **p < 0.01, ***p < 0.001. The treatments followed the strategy illustrated in **a** using 25 µM NVB or 2.5 pmol RPA, respectively

We hypothesized that inhibiting the MMEJ pathway, which competes with HDR for repairing DSBs, could improve HDR efficiency. To test this hypothesis, we established an hPSC line containing a mutant GFP transgene that can be rescued to express wild-type GFP through HDR mediated by CRISPR-Cas9 (Fig. 4a). We treated the cells with 25 µM NVB for 24 h before and after electroporation of the Cas9/sgRNA RNP and an ssODN donor and observed a significant increase in HDR efficiency compared to the control (Fig. 4b; Additional file 1: Fig. S4a-c). We also investigated the effect of recombinant RPA on HDR efficiency and found that low doses (less than 10 pmol) of RPA, premixed with the Cas9/sgRNA RNP and ssODN donor before electroporation, improved HDR efficiency (Fig. 4b; Additional file 1: Fig. S4a-c). However, higher doses of RPA diminished HDR efficiency, possibly due to dose-dependent interference with ssODN delivery into cells. This was demonstrated by FACS analysis of Cy3-labeled ssODN after co-electroporation with Cas9/sgRNA RNP and varying doses of RPA (Additional file 1: Fig. S4d). To validate this strategy in clinically relevant genes and/or cell types, we installed, via Cas9-mediated HDR, an *EPOR* gene mutation (G6002A) that can cause benign human erythrocytosis [45] in both human ESCs and primary peripheral blood erythroid progenitors and an activating *WAS* mutation (T882C) that is associated with X-linked neutropenia [46]. The findings demonstrated a consistent enhancement of HDR efficiency in the editing of both genes in different cell types when treated with NVB or using recombinant RPA, compared to the control group (Fig. 4c, d; Additional file 1: Fig. S4e). Hence, these data demonstrate that modulating POLQ activity and RPA level can increase HDR efficiency for precise genome editing.

## Discussion

A previous study reported a high frequency of MHs at LD breakpoint junctions induced by dual paired Cas9^D10A^ nickases or paired Cas9 nuclease [15]. Here, we also observed a similar phenomenon induced by a single Cas9 cut. However, the mechanism behind the phenomenon was not thoroughly understood. Increasing evidence has shown that MMEJ is not only a backup repair pathway but also actively functions when HR and NHEJ are intact [23, 47]. That CRISPR-Cas9 continuously recuts the target after error-free DNA repair (which regenerates the target) could increase the chance for LD-prone repair through the MMEJ pathway. Although how asymmetrical release of the 3’ end of non-target DNA strand after Cas9 cleavage and long-term residence of Cas9 on the broken ends of DNA [48] affect DNA repair pathway choice is unclear, that PARP1 knockdown does not affect LD frequency suggests CRISPR-Cas9-induced DSBs initiate MMEJ repair pathway via a PARP1-independent manner (Additional file 1: Fig. S2c). Although LIG3 is a predominant ligase of the MMEJ pathway that seals the nicks in DNA, its function could be replaced by other ligases, such as LIG1 [22]. Moreover, PARP1 and LIG3 deficiency did not affect LDs in Cas9-edited mouse ESCs [14]. POLQ plays a central role in MMEJ in higher organisms [23]. Knocking down or inhibiting POLQ caused a significant reduction of LDs, which suggested limited functional redundancy between POLQ and other DNA polymerases and reaffirmed the central role of MMEJ in Cas9-induced LD. RPA is involved in DNA replication and repair. We discovered that RPA deficiency led to more frequent LDs induced by CRISPR-Cas9, potentially because RPA prevents the annealing of resected ssDNA at MHs.

An increasing number of studies have been conducted on CRISPR-Cas9-induced LDs. One study showed that LD can be prevented by enhanced homology-directed repair (HDR) via the delivery of ssODNs or adeno-associated virus (AAV) donors and NHEJ mediated dsODN insertion in primary T cells and hematopoietic stem cells but not in iPSCs [11]. It can be construed as evidence that suppression of MMEJ via promoting the HDR and NHEJ pathways could reduce LD, which complements our direct mechanistic insights into MMEJ. Another study targeted 32 DNA repair genes associated with the NHEJ, MMEJ, and HR repair pathways in mouse ESCs and found that the NHEJ pathway hindered LD while the MMEJ pathway promoted LD [14], which is consistent with our results. However, *RPA*, a key player in LD discovered in our study, was not included in the 32 genes. A recent study showed that LDs and translocations can be reduced in T cells by the fusion of Cas9 with an optimized exonuclease TREX2, which prevents perfect DNA repair [49]. Although these tools are promising, they do not provide new insights to understand LDs. CRISPR-Cas engineering strategies such as base editor and primer editor rely on single-strand nicks to perform precise editing [13]; it will be of interest to apply the strategies of this study to understand whether these genome editing tools incur LDs and if MMEJ plays a similar role.

In this study, we first discovered that two key MMEJ genes (*POLQ* and *RPA*) regulate CRISPR-Cas9-induced LD formation and provided a mechanistic understanding of Cas9-induced LD. We then demonstrated that small-molecule inhibition of POLQ or supplying recombinant RPA together with Cas9/sgRNA RNP and ssODN can significantly increase HDR in hPSCs. Small molecule inhibitors are promising tools for improving the outcome of CRISPR-Cas editing. Recently, ART588, a potent and specific POLQ inhibitor, was reported to prevent CRISPR-Cas9-induced LDs and enhance HDR when combined with the NHEJ inhibitor NU7741 [50]. Similarly, another study found that inhibiting NHEJ and MMEJ can enhance precise genome editing in a chemical screen [51]. These studies are consistent with our results and further underscore the critical role of MMEJ in LDs. Interestingly, a previous study showed that NVB treatment did not improve HDR in human primary T cells [52]. We note that the treatment regime and cell type used therein are different from this study, suggesting the choice of DNA repair pathway may be complex and context dependent. In line with our findings, another study showed that POLQ antagonizes RPA to promote MMEJ and suppresses CRISPR-Cas9-mediated HDR [53]. Moreover, POLQ deficiency does not affect the genetic stability and development of *P. patens* [54]. Thus, small-molecule inhibition of POLQ and/or delivery of recombinant RPA offers a simple, convenient, and potentially safe way to reduce the risk of the unwanted LDs and improve HDR efficiency.

## Conclusions

We demonstrate that the MMEJ pathway plays an important role in CRISPR-Cas9-induced LD and find two key MMEJ genes (*POLQ* and *RPA*) perform oppositely in CRISPR-Cas9-induced LD and HDR. We provide a potentially safe strategy to decrease the CRISPR-Cas9-induced LD and increase HDR efficiency by modulating POLQ and RPA. The strategy presented in this study may help improve the safety and efficacy of CRISPR therapy, the first of which targeting sickle cell disease approved by the U.K. Medicines and Healthcare products Regulatory Agency (MHRA) and the U.S. Food and Drug Administration (FDA) in 2023 [55].

## Methods

### Cell culture

The H1 hESC line was purchased from WiCell Institute. The H1-iCas9 ESC line is a gift from Danwei Huangfu’s laboratory. The wild-type iPSC line was reprogrammed and well characterized in previous studies [44, 56, 57]. The study was approved by the KAUST Institutional Biosafety and Bioethics Committee (IBEC). All hPSCs were cultured in Essential 8 medium (ThermoFisher, Cat# A1517001) in rhLaminin-521 (ThermoFisher, Cat# A29249) coated wells with medium change daily. The peripheral blood mononuclear cells were isolated from the whole blood of a healthy donor via a standard Ficoll-Paque-based protocol and further cultured in StemSpan™-ACF Erythroid Expansion medium (STEMCELL Technology, Cat# 09860) for 13 days with medium change every 3 days to expand the erythroid progenitors. The erythroid progenitors were analyzed by FACS before CRISPR-Cas9 editing.

### Plasmids and lentiviral packaging

Oligonucleotides containing the gRNA sequence were annealed and subsequently inserted into a lentiGuide-puro plasmid (Addgene Cat # 52963) following a published protocol [58]. The full-length RPA including the open reading frames (ORF) of RPA1, RPA2, and RPA3 were cloned from cDNA of H1 ESCs, and the GFP ORF was cloned from pInducer21 (Addgene, Cat # 46948). Subsequently, the ORFs of RPA and GFP were inserted into pInducer21 using the Gateway cloning method. The sequences were confirmed by Sanger sequencing. The gRNA lentiGuide-puro, newly constructed vectors, and pEGIP*35 (Addgene, Cat# 26,776) were packaged into lentiviruses individually. Briefly, the plasmid was premixed with packaging vectors and then transfected into HEK293T cells using Lipofectamine 3000 (ThermoFisher, Cat# L3000015). The lentivirus was harvested two times after 48 h and 72 h. The lentivirus was concentrated with PEG-it Virus Precipitation Solution (System Biosciences, Cat# LV810A-1/ LV825A-1) and stored in a –80 °C freezer.

### siRNA transfection

The protocol of esiRNA transfection was adapted to the instruction of Lipofectamine RNAiMAX reagent (ThermoFisher, Cat# 13778150). H1-iCas9 cells were harvested after treatment with 10 µM Y-27632 (Abcam, Cat# ab120129) for 1 h. The esiRNA/RNAiMAX solution was prepared for 3 wells per siRNA in a 12-well format plate as the following procedure: Mix 1 was prepared by adding 13.5 µl RNAiMAX reagent to 225 µl opti-MEM and vortexing for a few seconds. Mix 2 was prepared by adding 90 pmol esiRNA to 225 µl opti-MEM and pipetting a few times. The esiRNA/RNAiMAX solution was prepared by adding Mix2 into Mix1 and incubating for 5 min, after which the mixture was used to resuspend a 1.5 million cell pellet. After 30 min incubation with esiRNA/RNAiMAX solution, the cells were aliquoted equally into 3 rhLaminin-521 coated wells and cultured in 37°C, 5% CO2 incubator. The cell samples were collected after 24 h for knockdown efficiency analysis.

### Quantitative PCR (qPCR)

The RNA was extracted using an rNeasy Mini kit (Qiagen, Cat #74106) and reverse transcribed to cDNA using iScript Reverse Transcription Supermix (BioRad, Cat# 1708840). The qPCR was performed on a CFX384 real-time PCR detection system (BioRad) using SsoAdvanced Universal SYBR Green Supermix (BioRad, Cat# 725270). The qPCR primers were shown in Additional file 2: Table S1 [35].

### Droplet digital PCR (ddPCR)

The genomic DNA was extracted after 3 days post-electroporation using a DNeasy Blood & Tissue kit (Qiagen, Cat #69506) and quantified with a Qubit instrument. The ddPCR was performed on a Bio-Rad QX200 system using ddPCR Supermix for Probes (No dUTP) (Bio-Rad, Cat #1,863,024) following the manufacturer’s protocols. The 20 × assay mixture was comprised of 18 µM each primer and 5 µM each probe. One reaction contains 5 ng genomic DNA, 1 × assay mix, and 1 × ddPCR Supermix. The probes and oligos were shown in Additional file 2: Table S1.

### Flow cytometry

For *PIGA* gene edited samples, the gRNA lentivirus infected H1-iCas9 cells were treated with 2 µg/ml doxycycline for 2 days to induce Cas9 expression for gene editing. After the doxycycline treatment for 10 days, the cells were harvested and washed twice with PBS buffer containing 3% BSA and filtered through a 70-µm strainer. For each sample, 100,000 cells were stained with 2 µl FLAER Alexa488 (Cederlane, Cat# NC9870611) in 100 µl PBS buffer containing 3% BSA for 15 min at room temperature. The stained cells were washed once with PBS buffer containing 3% BSA and resuspended in 200 µl FACS buffer containing 1 µg/ml DAPI for FACS analysis using a BD FACSAria™ Fusion cytometer. For *CD9* gene edited samples, cells were harvested and washed once in FACS buffer with 2% FBS. Subsequently, 10,000 cells were stained in 100 µl FACS buffer with 2% FBS and 1 µl PE anti-CD9 (BioLegend, Cat# 312,106) for 30 min at 4°C, followed by two washes with FACS buffer containing 2% FBS. For GFPmut correction samples, the cells were harvested after 3 days post-electroporation and passed through a 70-µm strainer. The cells were resuspended in 200 µl FACS buffer containing 1 µg/ml DAPI and loaded onto a BD FACSAria™ Fusion cytometer for analysis. For *LAMP2* gene edited samples, a manufacturer’s protocol of BD Cytofix/Cytoperm™ Fixation/Permeabilization Kit (BD, Cat# 554714) was followed. Briefly, the cells were fixed in Fixation/Permeabilization solution at 4°C for 20 min and washed twice in BD Perm/Wash™ Buffer, followed by staining using 50 µl BD Perm/Wash™ Buffer containing 2 µl FITC anti-LAMP2 (eBioscience, Cat# 11–1078-42) for 30 min at 4°C. After two washes, the cells were resuspended in FACS buffer and analyzed using a BD FACSymphony™ A3 Cell Analyzer.

### Cell cycle synchronization and analysis

The cell cycle synchronization protocol was adapted from a previous publication [36]. In brief, PIGA intr5_1 gRNA positive H1-iCas9 ESCs were seeded at a density of 2 × 10^5^ cells per well in a 12-well plate. To synchronize the cells at the G2/M phase, a 16-h treatment with 100 ng/ml nocodazole (Abcam, Cat# ab120630) was administered. Subsequently, the cells were washed twice with prewarmed 1 × PBS and then cultured in fresh E8 medium for 4 h or 12 h to release cells in the G1 or S phase, respectively. Alternatively, the synchronized cells were treated with 2 µg/ml doxycycline to induce Cas9 expression and genome editing, followed by 10 days’ culture for LD analysis.

Cell cycle analysis was performed using a standard protocol. Initially, the cell pellets were fixed by adding cold 70% ethanol dropwise while vortexing and then incubated overnight at − 20°C. Subsequently, the cells were washed twice and resuspended in FACS buffer containing 200 µg/ml rNase. After a 20-min incubation at room temperature, the cells were washed once and resuspended in FACS buffer containing 1 µg/ml propidium iodide (PI). Following a 10-min incubation at room temperature, the samples were ready for FACS analysis.

### CRISPR-Cas9 genome editing

WT and Hifi-Cas9 were purchased from IDT. The gRNAs used in this study were designed using Benchling (https://www.benchling.com/crispr) and their sequences were shown in Additional file 2: Table S1 [6]. The gRNAs were obtained either through in vitro transcribed by a MEGAshortscript™ T7 Transcription kit (ThermoFisher, Cat# AM1354) or ordered through IDT as Alt-R crRNAs or sgRNAs. For each electroporation, 50 pmol of Alt-R gRNA and 50 pmol of Cas9 were mixed and incubated at room temperature for 10 min to form ribonucleoprotein (RNP). Buffer R (from the Neon system kit) was added to the RNP to a final volume of 10 µl; 200,000 single cells were electroporated using a Neon system (ThermoFisher) with the setting of 1600 V, 10 ms width and 3 pulses. For the HDR study, 30 pmol ssODN was mixed with 50 pmol RNP before the electroporation. The cells were seeded in 1 well of a 24-well plate immediately after electroporation.

### PacBio and Nanopore sequencing

The genomic DNA of edited cells was extracted using a Blood & Tissue Kit (Qiagen, Cat# 69506). The UMI labeling was performed following a published protocol [7]. Briefly, the target locus was labeled by one-cycle PCR using a UMI primer (Additional file 2: Table S1) in a 25 µl reaction including 50 ng genomic DNA, 1 µM UMI primer (containing a universal forward primer sequence, 10 nts UMI barcode, and a target locus forward primer sequence, see it in Additional file 2: Table S1), 12.5 µl 2 × Platinum SuperFi PCR Master Mix (ThermoFisher, Cat# 12358010), following the program: initial denaturation at 98°C for 70 s, gradient annealing from 70°C to 65°C with 1°C /5 s ramp rate, extension at 72°C for 7 min, and holding at 4°C. The UMI labeled DNA was purified by 0.8 × AMPure XP beads, then mixed with a universal forward primer, a target locus reverse primer (Additional file 2: Table S1), and PrimeSTAR GXL DNA polymerase (Takara, Cat# R050A), and amplified following the program: initial denaturation at 95 °C for 2 min, 98 °C for 10 s, 68 °C for 7 min for 30 cycles, 68 °C for 5min, and hold at 4 °C. For bulk ONT nanopore sequencing, the amplicons were amplified using the target locus forward and reverse primers (Additional file 2: Table S1). The amplicons were purified with AMPure XP beads and used for PacBio or Nanopore library preparation.

For Nanopore sequencing, the library preparation was done using the ligation sequencing kit (Oxford Nanopore Technologies, Cat# SQK-LSK109) following its standard protocol. The Nanopore sequencing was performed on an Oxford Nanopore MinION sequencer using R9.4.1 flow cells. The reads were base called using Guppy basecaller (v5.0.7). Library preparations of PacBio sequencing were performed with the Sequel Sequencing Kit 3.0 and loaded on the PacBio Sequel instrument with SMRT Cell 1 M v3 LR Tray. PacBio official tool termed ccs (v3.4.1) was used to generate HiFi Reads. All procedures were performed according to the manufacturer’s protocols.

Data analysis was performed using VAULT as described previously [7]. In brief, the UMI primer sequence, fastq file, and reference amplicon sequence were provided to the algorithm. VAULT will extract mappable reads followed by extraction of UMI sequences from reads. Reads will then be grouped based on their UMI sequences and used for parallel analysis of SNVs and SVs. The “vault summarize” command was used to generate the analysis summary. For bulk ONT Nanopore and PacBio sequencing, the reads were aligned to the hg38 reference genome by minimap2 (v2.11) to check for large deletions. The large deletion frequency was calculated as the percentage of deletion-containing reads from alignment results.

### Recombinant human RPA protein

Human RPA was expressed and purified as described previously [59, 60]. Briefly, the cloned plasmid was transformed into BL21 (DE3) *E. coli*. The cells were grown in 2YT media at 37 °C to an OD_600_ of 0.7 and protein expression was induced with 0.5 mM IPTG and further incubated for 4–6 h at 37 °C. The cells were collected by centrifugation and lysed by lysozyme and sonication. The supernatant was loaded onto a HisTrap HP 5 ml column (Cytiva) followed by the HiTrap Blue affinity column (Cytiva). RPA fractions containing all subunits were concentrated and loaded onto a HiLoad 16/600 Superdex 200 pg column (Cytiva). RPA protein fractions were flash-frozen and stored at − 80 °C.

### Statistical analysis

The data in the figures are shown as the mean ± SD unless indicated otherwise. Comparisons were performed with two-sided Student’s *t*-test unless indicated otherwise.

### Supplementary Information

**Additional file 1: Fig. S1.** CRISPR-Cas9 genome editing induced LD in human pluripotent stem cells. **Fig. S2.** Modulation of MMEJ can regulate CRISPR-induced LD frequency. **Fig. S2.** Modulation of MMEJ regulates CRISPR-induced LD frequency. **Fig. S4.** Modulation of MMEJ improves HDR efficiency.

**Additional file 2**: **Table S1**. Oligonucleotide information used in this study

**Additional file 3**: Original blots and gels.

## Data Availability

All data generated or analyzed during this study are included in this published article, its supplementary information files, and publicly available repositories. Raw sequencing data are available in the SRA database (accession ID PRJNA953777), which are accessible with the following link: https://dataview.ncbi.nlm.nih.gov/object/PRJNA953777.

## Abbreviations

Cas9: CRISPR-associated protein 9
CRISPR: Clustered regularly interspaced short palindromic repeats
ddPCR: Droplet digital PCR
DSB: Double strand break
ESC: Embryonic stem cell
FACS: Fluorescence-activated cell sorting
HDR: Homology-directed repair
hPSC: Human pluripotent stem cell
HR: Homologous recombination
IDMseq: Long-read individual-molecule sequencing
Indels: Insertions and deletions
iPSC: Induced pluripotent stem cell
LD: Large deletion
LIG3: DNA ligase 3
MH: Microhomology
MMEJ: Microhomology-mediated end joining
NHEJ: Non-homologous end joining
NVB: Novobiocin
PARP1: Genes-poly [ADP-ribose] polymerase 1
POLQ: DNA polymerase theta
qPCR: Quantitative PCR
RNP: Ribonucleoprotein
RPA: Replication protein A
sgRNA: Single-guide RNA
SSA: Single-stranded annealing
ssODN: Single-stranded oligodeoxynucleotide
SV: Structural variation

## References

[1] Chakrabarti AM, Henser-Brownhill T, Monserrat J, Poetsch AR, Luscombe NM, Scaffidi P (2019). Target-specific precision of CRISPR-mediated genome editing. Mol Cell.

[2] Guo T, Feng YL, Xiao JJ, Liu Q, Sun XN, Xiang JF, Kong N, Liu SC, Chen GQ, Wang Y (2018). Harnessing accurate non-homologous end joining for efficient precise deletion in CRISPR/Cas9-mediated genome editing. Genome Biol.

[3] Koike-Yusa H, Li Y, Tan EP, Velasco-Herrera Mdel C, Yusa K (2014). Genome-wide recessive genetic screening in mammalian cells with a lentiviral CRISPR-guide RNA library. Nat Biotechnol.

[4] van Overbeek M, Capurso D, Carter MM, Thompson MS, Frias E, Russ C, Reece-Hoyes JS, Nye C, Gradia S, Vidal B (2016). DNA repair profiling reveals nonrandom outcomes at Cas9-mediated breaks. Mol Cell.

[5] Tan EP, Li Y, Velasco-Herrera Mdel C, Yusa K, Bradley A (2015). Off-target assessment of CRISPR-Cas9 guiding RNAs in human iPS and mouse ES cells. Genesis.

[6] Kosicki M, Tomberg K, Bradley A (2018). Repair of double-strand breaks induced by CRISPR-Cas9 leads to large deletions and complex rearrangements. Nat Biotechnol.

[7] Bi C, Wang L, Yuan B, Zhou X, Li Y, Wang S, Pang Y, Gao X, Huang Y, Li M (2020). Long-read individual-molecule sequencing reveals CRISPR-induced genetic heterogeneity in human ESCs. Genome Biol.

[8] Adikusuma F, Piltz S, Corbett MA, Turvey M, McColl SR, Helbig KJ, Beard MR, Hughes J, Pomerantz RT, Thomas PQ (2018). Large deletions induced by Cas9 cleavage. Nature.

[9] Zuccaro MV, Xu J, Mitchell C, Marin D, Zimmerman R, Rana B, Weinstein E, King RT, Palmerola KL, Smith ME (2020). Allele-specific chromosome removal after Cas9 cleavage in human embryos. Cell.

[10] Alanis-Lobato G, Zohren J, McCarthy A, Fogarty NME, Kubikova N, Hardman E, Greco M, Wells D, Turner JMA, Niakan KK (2021). Frequent loss of heterozygosity in CRISPR-Cas9-edited early human embryos. Proc Natl Acad Sci U S A.

[11] Wen W, Quan ZJ, Li SA, Yang ZX, Fu YW, Zhang F, Li GH, Zhao M, Yin MD, Xu J (2021). Effective control of large deletions after double-strand breaks by homology-directed repair and dsODN insertion. Genome Biol.

[12] Hoijer I, Emmanouilidou A, Ostlund R, van Schendel R, Bozorgpana S, Tijsterman M, Feuk L, Gyllensten U, den Hoed M, Ameur A (2022). CRISPR-Cas9 induces large structural variants at on-target and off-target sites in vivo that segregate across generations. Nat Commun.

[13] Song Y, Liu Z, Zhang Y, Chen M, Sui T, Lai L, Li Z (2020). Large-fragment deletions induced by Cas9 cleavage while not in the BEs system. Mol Ther Nucleic Acids.

[14] Kosicki M, Allen F, Steward F, Tomberg K, Pan Y, Bradley A (2022). Cas9-induced large deletions and small indels are controlled in a convergent fashion. Nat Commun.

[15] Owens DDG, Caulder A, Frontera V, Harman JR, Allan AJ, Bucakci A, Greder L, Codner GF, Hublitz P, McHugh PJ (2019). Microhomologies are prevalent at Cas9-induced larger deletions. Nucleic Acids Res.

[16] Wang M, Wu W, Wu W, Rosidi B, Zhang L, Wang H, Iliakis G (2006). PARP-1 and Ku compete for repair of DNA double strand breaks by distinct NHEJ pathways. Nucleic Acids Res.

[17] Luedeman ME, Stroik S, Feng W, Luthman AJ, Gupta GP, Ramsden DA (2022). Poly(ADP) ribose polymerase promotes DNA polymerase theta-mediated end joining by activation of end resection. Nat Commun.

[18] Mimitou EP, Symington LS (2009). Nucleases and helicases take center stage in homologous recombination. Trends Biochem Sci.

[19] San Filippo J, Sung P, Klein H (2008). Mechanism of eukaryotic homologous recombination. Annu Rev Biochem.

[20] McVey M (2014). RPA puts the brakes on MMEJ. Nat Struct Mol Biol.

[21] Kent T, Chandramouly G, McDevitt SM, Ozdemir AY, Pomerantz RT (2015). Mechanism of microhomology-mediated end-joining promoted by human DNA polymerase theta. Nat Struct Mol Biol.

[22] Wang H, Xu X (2017). Microhomology-mediated end joining: new players join the team. Cell Biosci.

[23] Sfeir A, Symington LS (2015). Microhomology-mediated end joining: a back-up survival mechanism or dedicated pathway?. Trends Biochem Sci.

[24] Audebert M, Salles B, Calsou P (2004). Involvement of poly(ADP-ribose) polymerase-1 and XRCC1/DNA ligase III in an alternative route for DNA double-strand breaks rejoining. J Biol Chem.

[25] Boutin J, Rosier J, Cappellen D, Prat F, Toutain J, Pennamen P, Bouron J, Rooryck C, Merlio JP, Lamrissi-Garcia I (2021). CRISPR-Cas9 globin editing can induce megabase-scale copy-neutral losses of heterozygosity in hematopoietic cells. Nat Commun.

[26] Papathanasiou S, Markoulaki S, Blaine LJ, Leibowitz ML, Zhang CZ, Jaenisch R, Pellman D (2021). Whole chromosome loss and genomic instability in mouse embryos after CRISPR-Cas9 genome editing. Nat Commun.

[27] Shi ZD, Lee K, Yang D, Amin S, Verma N, Li QV, Zhu Z, Soh CL, Kumar R, Evans T (2017). Genome editing in hPSCs reveals GATA6 haploinsufficiency and a genetic interaction with GATA4 in human pancreatic development. Cell Stem Cell.

[28] Tsuchida CA, Brandes N, Bueno R, Trinidad M, Mazumder T, Yu B, Hwang B, Chang C, Liu J, Sun Y (2023). Mitigation of chromosome loss in clinical CRISPR-Cas9-engineered T cells. Cell.

[29] Cullot G, Boutin J, Toutain J, Prat F, Pennamen P, Rooryck C, Teichmann M, Rousseau E, Lamrissi-Garcia I, Guyonnet-Duperat V (2019). CRISPR-Cas9 genome editing induces megabase-scale chromosomal truncations. Nat Commun.

[30] Rayner E, Durin MA, Thomas R, Moralli D, O'Cathail SM, Tomlinson I, Green CM, Lewis A (2019). CRISPR-Cas9 causes chromosomal instability and rearrangements in cancer cell lines, detectable by cytogenetic methods. CRISPR J.

[31] Przewrocka J, Rowan A, Rosenthal R, Kanu N, Swanton C (2020). Unintended on-target chromosomal instability following CRISPR/Cas9 single gene targeting. Ann Oncol.

[32] Brunet E, Jasin M (2018). Induction of chromosomal translocations with CRISPR-Cas9 and other nucleases: understanding the repair mechanisms that give rise to translocations. Adv Exp Med Biol.

[33] Hunt JMT, Samson CA, Rand AD, Sheppard HM (2023). Unintended CRISPR-Cas9 editing outcomes: a review of the detection and prevalence of structural variants generated by gene-editing in human cells. Hum Genet.

[34] Beying N, Schmidt C, Pacher M, Houben A, Puchta H (2020). CRISPR-Cas9-mediated induction of heritable chromosomal translocations in Arabidopsis. Nat Plants.

[35] Quilter CR, Karcanias AC, Bagga MR, Duncan S, Murray A, Conway GS, Sargent CA, Affara NA (2010). Analysis of X chromosome genomic DNA sequence copy number variation associated with premature ovarian failure (POF). Hum Reprod.

[36] Yiangou L, Grandy RA, Morell CM, Tomaz RA, Osnato A, Kadiwala J, Muraro D, Garcia-Bernardo J, Nakanoh S, Bernard WG (2019). Method to synchronize cell cycle of human pluripotent stem cells without affecting their fundamental characteristics. Stem Cell Reports.

[37] Kawamura K, Bahar R, Seimiya M, Chiyo M, Wada A, Okada S, Hatano M, Tokuhisa T, Kimura H, Watanabe S (2004). DNA polymerase theta is preferentially expressed in lymphoid tissues and upregulated in human cancers. Int J Cancer.

[38] Schrempf A, Slyskova J, Loizou JI (2021). Targeting the DNA repair enzyme polymerase theta in cancer therapy. Trends Cancer.

[39] Zhou J, Gelot C, Pantelidou C, Li A, Yucel H, Davis RE, Farkkila A, Kochupurakkal B, Syed A, Shapiro GI (2021). A first-in-class polymerase theta inhibitor selectively targets homologous-recombination-deficient tumors. Nat Cancer.

[40] Lemee F, Bergoglio V, Fernandez-Vidal A, Machado-Silva A, Pillaire MJ, Bieth A, Gentil C, Baker L, Martin AL, Leduc C (2010). DNA polymerase theta up-regulation is associated with poor survival in breast cancer, perturbs DNA replication, and promotes genetic instability. Proc Natl Acad Sci U S A.

[41] Allera-Moreau C, Rouquette I, Lepage B, Oumouhou N, Walschaerts M, Leconte E, Schilling V, Gordien K, Brouchet L, Delisle MB (2012). DNA replication stress response involving PLK1, CDC6, POLQ, RAD51 and CLASPIN upregulation prognoses the outcome of early/mid-stage non-small cell lung cancer patients. Oncogenesis.

[42] Zatreanu D, Robinson HMR, Alkhatib O, Boursier M, Finch H, Geo L, Grande D, Grinkevich V, Heald RA, Langdon S (2021). Poltheta inhibitors elicit BRCA-gene synthetic lethality and target PARP inhibitor resistance. Nat Commun.

[43] Deng SK, Gibb B, de Almeida MJ, Greene EC, Symington LS (2014). RPA antagonizes microhomology-mediated repair of DNA double-strand breaks. Nat Struct Mol Biol.

[44] Kreitzer FR, Salomonis N, Sheehan A, Huang M, Park JS, Spindler MJ, Lizarraga P, Weiss WA, So PL, Conklin BR (2013). A robust method to derive functional neural crest cells from human pluripotent stem cells. Am J Stem Cells.

[45] de la Chapelle A, Traskelin AL, Juvonen E (1993). Truncated erythropoietin receptor causes dominantly inherited benign human erythrocytosis. Proc Natl Acad Sci U S A.

[46] Beel K, Cotter MM, Blatny J, Bond J, Lucas G, Green F, Vanduppen V, Leung DW, Rooney S, Smith OP (2009). A large kindred with X-linked neutropenia with an I294T mutation of the Wiskott-Aldrich syndrome gene. Brit J Haematol.

[47] Truong LN, Li Y, Shi LZ, Hwang PY, He J, Wang H, Razavian N, Berns MW, Wu X (2013). Microhomology-mediated end joining and homologous recombination share the initial end resection step to repair DNA double-strand breaks in mammalian cells. Proc Natl Acad Sci U S A.

[48] Richardson CD, Ray GJ, DeWitt MA, Curie GL, Corn JE (2016). Enhancing homology-directed genome editing by catalytically active and inactive CRISPR-Cas9 using asymmetric donor DNA. Nat Biotechnol.

[49] Yin J, Lu R, Xin C, Wang Y, Ling X, Li D, Zhang W, Liu M, Xie W, Kong L (2022). Cas9 exo-endonuclease eliminates chromosomal translocations during genome editing. Nat Commun.

[50] Schimmel J, Munoz-Subirana N, Kool H, van Schendel R, van der Vlies S, Kamp JA, de Vrij F, Kushner SA, Smith GCM, Boulton SJ (2023). Modulating mutational outcomes and improving precise gene editing at CRISPR-Cas9-induced breaks by chemical inhibition of end-joining pathways. Cell Rep.

[51] Wimberger S, Akrap N, Firth M, Brengdahl J, Engberg S, Schwinn MK, Slater MR, Lundin A, Hsieh PP, Li S (2023). Simultaneous inhibition of DNA-PK and Polϴ improves integration efficiency and precision of genome editing. Nat Commun.

[52] Shy BR, Vykunta VS, Ha A, Talbot A, Roth TL, Nguyen DN, Pfeifer WG, Chen YY, Blaeschke F, Shifrut E, et al. High-yield genome engineering in primary cells using a hybrid ssDNA repair template and small-molecule cocktails. Nat Biotechnol 2023;41(4):521–31. 10.1038/s41587-022-01418-8 PMC10065198 36008610

[53] Mateos-Gomez PA, Kent T, Deng SK, McDevitt S, Kashkina E, Hoang TM, Pomerantz RT, Sfeir A (2017). The helicase domain of Poltheta counteracts RPA to promote alt-NHEJ. Nat Struct Mol Biol.

[54] Mara K, Charlot F, Guyon-Debast A, Schaefer DG, Collonnier C, Grelon M, Nogue F (2019). POLQ plays a key role in the repair of CRISPR/Cas9-induced double-stranded breaks in the moss Physcomitrella patens. New Phytol.

[55] Sheridan C (2024). The world's first CRISPR therapy is approved: who will receive it?. Nat Biotechnol.

[56] Li M, Suzuki K, Qu J, Saini P, Dubova I, Yi F, Lee J, Sancho-Martinez I, Liu GH, Izpisua Belmonte JC (2011). Efficient correction of hemoglobinopathy-causing mutations by homologous recombination in integration-free patient iPSCs. Cell Res.

[57] Suzuki K, Yu C, Qu J, Li M, Yao X, Yuan T, Goebl A, Tang S, Ren R, Aizawa E (2014). Targeted gene correction minimally impacts whole-genome mutational load in human-disease-specific induced pluripotent stem cell clones. Cell Stem Cell.

[58] Sanjana NE, Shalem O, Zhang F (2014). Improved vectors and genome-wide libraries for CRISPR screening. Nat Methods.

[59] Lancey C, Tehseen M, Raducanu VS, Rashid F, Merino N, Ragan TJ, Savva CG, Zaher MS, Shirbini A, Blanco FJ (2020). Structure of the processive human Pol delta holoenzyme. Nat Commun.

[60] Henricksen LA, Umbricht CB, Wold MS (1994). Recombinant replication protein A: expression, complex formation, and functional characterization. J Biol Chem.

