# Supplementary material for: Modulation of the microhomology-mediated end joining pathway suppresses large deletions and enhances homology-directed repair following CRISPR-Cas9-induced DNA breaks
Source: BMC Biol. 2024 Apr 29;22:101. doi: 10.1186/s12915-024-01896-z (PMC11059712; doi:10.1186/s12915-024-01896-z)
Supplement: Supplementary file 1 — Additional file 1: Fig. S1. CRISPR-Cas9 genome editing induced LD in human pluripotent stem cells. Fig. S2. Modulation of MMEJ can regulate CRISPR-induced LD frequency. Fig. S2. Modulation of MMEJ regulates CRISPR-induced LD frequency. Fig. S4. Modulation of MMEJ improves HDR efficiency. [file 12915_2024_1896_MOESM1_ESM.docx]

**Modulation of the microhomology-mediated end joining pathway suppresses large deletions and enhances homology-directed repair following CRISPR-Cas9-induced DNA breaks**

**Supplementary Figures and Figure Legends**

**Fig. S1 CRISPR-Cas9 genome editing induced LD in human pluripotent stem cells**

**a** Agarose gel electrophoresis of long-range PCR products of SH2B3 and H1.3 edited cell lines, the red colored sample indicate LD clone.

**b** Schematic of the strategy to analyze CRISPR-induced LDs in *PIGA* locus (created with BioRender.com).

**c** Representative Integrative Genomics Viewer (IGV) tracks and coverage of long-read sequencing data on the PIGA locus from PIGA FLAER positive and negative sorted populations. The dashed arrow indicates the position of the PIGA FLAER positive LD proximal end on the PIGA locus. The scissor indicates the CRISPR/Cas9 cutting site.

**d** MH frequency in deletions ≥ 30 bp of PIGA intr1_1 sgRNA quantified from long-read sequencing data. **** *p* < 0.0001, Fisher’s exact test.

**e** Top: the location of PIGA gRNAs in the PIGA genomic locus; bottom left: example flow cytometry analysis of PIGA expression using the FLAER assay; bottom right: LD frequency of PIGA gRNAs screened, biological replicates *n* = 3.

**f** Plot of the LD frequency and the distance between the sgRNA and its nearest exon.

**g** Top: schematic of the target gene loci on X chromosome; bottom left: normalized copy number of target genes, biological replicates *n* = 3; bottom right: copy number measurements performed by ddPCR for *WAS* gen*e*, this experiment was performed more than three biological replicates, ns: not significant.

**Fig. S2 Modulation of MMEJ can regulate CRISPR-induced LD frequency.**

**a** Flow cytometry analysis of PIGA expression, the number in the gate indicates the percentage of PIGA FLAER negative population.

**b** Representative Western blotting analysis for RPA1 and POLQ expression. The grey value was quantified using ImageJ and normalized by the control siRNA (siCtrl) treated sample.

**c** Left: relative mRNA level of siRNA target genes, LIG3 and PARP1; right LD frequency quantified by FACS, biological replicates *n* = 3. **** *p* < 0.0001, ns: not significant.

**d** Schematic of the strategy and workflow for cell cycle synchronization by nocodazole and LD analysis by FACS (created with BioRender.com).

**e** Cell cycle analysis following nocodazole treatment strategy by FACS, the percentage of cell phase was calculated by the Watson pragmatic fitting algorithm, biological replicates *n* = 3.

**f** LD frequency of different doxycycline exposure time in H1-iCas9 PIGA intr5_1 ESCs, quantified by FACS, biological replicates *n* = 6, ns: not significant.

**g** LD frequency of cell cycle synchronized cells quantified by FACS, biological replicates *n* = 3 (each with three technical replicates).

**h** Cell cycle analysis of RPA and POLQ knockdown cells by FACS, the percentage of cell phase was calculated by the Watson pragmatic fitting algorithm, biological replicates *n* = 2 (each with two technical replicates).

**i** FACS analysis of pluripotency markers for NVB treated H1 hESCs.

**j** Live cell count of H1 hESCs at 24 hr and 48 hr after NVB treatment, biological replicates *n* = 3, ns: not significant.

**k** LD size distribution analysis for RPA and POLQ knockdown samples.

**l** MH frequency in LDs of RPA and POLQ knockdown samples. The numerator indicates the MH ≥ 2 bp event number, and the denominator indicates the LD event number detected by IDMseq. ns: not significant.

**m** LD size distribution analysis for NVB treated and RPA overexpression samples.

**n** MH frequency in LDs of NVB treated and RPA overexpression samples. The numerator indicates the MH ≥ 2 bp event number, and the denominator indicates the LD event number detected by IDMseq. ** *p* < 0.01, * *p* < 0.05, Fisher’s exact test.

**o** CRISPR-Cas9 editing efficiency using an exonic PIGA gRNA, ex2_1 sgRNA, quantified by FACS, biological replicates *n* = 3, ns: not significant.

**Fig. S3 Modulation of MMEJ regulates CRISPR-induced LD frequency.**

**a** Top: the location of CD9 gRNAs ; bottom: LD frequency of CD9 gRNAs screened, biological replicates *n* = 3.

**b-c** LD frequency quantified by FACS, biological replicates *n* = 3, ** p* < 0.05, *** p* < 0.01. OE: overexpression.

**d** Top: the location of LAMP2 intronic gRNAs, the numbers indicate the distances between sgRNA cutting sites and the nearest exons; bottom: example flow cytometry analysis of LAMP2 expression.

**e-g** LD frequency quantified by FACS, biological replicates *n*= 3, * *p* < 0.05, ** *p*< 0.01, **** p* < 0.001, ***** p* < 0.0001. OE: overexpression.

**h** Top: the location of the WAS intronic gRNA; bottom: frequency of LD (≥ 30 bp) quantified by ONT long-read sequencing. The numerator indicates the LD event number, and the denominator indicates the total event number detected by nanopore reads. ***** p* < 0.0001, Fisher’s exact test.

**i** Top: the location of the HBB intronic gRNA; bottom: frequency of LD (≥ 30 bp) quantified by ONT long-read sequencing. The numerator indicates the LD event number, and the denominator indicates the total event number detected by nanopore reads. ***** p* < 0.0001, Fisher’s exact test.

**Fig. S4 Modulation of MMEJ improves HDR efficiency.**

**a** Representative Coomassie-stained SDS-PAGE images of the RPA protein complex including the RPA1, RPA2 and RPA3 subunits, kDa: kilodalton.

**b** Flow cytometry analysis of GFP expression.

**c** GFP positive population quantified by FACS, biological replicates *n* = 3; * *p* < 0.05, ** *p* < 0.01, *** *p* < 0.001, **** *p* < 0.0001, ns: not significant.

**d** Delivery efficiency of Cy3-ssODN mixed with recombinant RPA.

**e** Flow cytometry analysis of human primary peripheral blood erythroid progenitor cell surface markers.
