# Supplementary material for: Modulation of the microhomology-mediated end joining pathway suppresses large deletions and enhances homology-directed repair following CRISPR-Cas9-induced DNA breaks
Source: BMC Biol. 2024 Apr 29;22:101. doi: 10.1186/s12915-024-01896-z (PMC11059712; doi:10.1186/s12915-024-01896-z)
Supplement: Supplementary file 2 — Additional file 2: Table S1. Oligonucleotide information used in this study [file 12915_2024_1896_MOESM2_ESM.docx]

**Table S1**

Oligonucleotides

| Names | Sequences (5’-3’) | References |
| --- | --- | --- |
| PIGA gRNA intr1_1 F | CACCGTGGTAAACCATGATATGCTG | [6] |
| PIGA gRNA intr1_1 R | aaacCAGCATATCATGGTTTACCAC |  |
| PIGA gRNA intr1_2 F | CACCGTTTAGGGCAAAATTAAGACA |  |
| PIGA gRNA intr1_2 R | aaacTGTCTTAATTTTGCCCTAAAC |  |
| PIGA gRNA intr2.e F | CACCGTCCAAGAAGAGCGATTTTTC |  |
| PIGA gRNA intr2.e R | aaacGAAAAATCGCTCTTCTTGGAC |  |
| PIGA gRNA intr2_1 F | CACCGATTAAAACATCAAAGGTTGG |  |
| PIGA gRNA intr2_1 R | aaacCCAACCTTTGATGTTTTAATC |  |
| PIGA gRNA intr2_2 F | CACCgctgaggcaggcagatcacg |  |
| PIGA gRNA intr2_2 R | aaaccgtgatctgcctgcctcagc |  |
| PIGA gRNA intr2_3 F | CACCGtgtagtcccagctactccgg |  |
| PIGA gRNA intr2_3 R | aaacccggagtagctgggactacaC |  |
| PIGA intr2_4 F | CACCGcagcaagcccattgatcgtg |  |
| PIGA gRNA intr2_4 R | aaaccacgatcaatgggcttgctgC |  |
| PIGA gRNA intr2_5 F | CACCGTGGAAGGTAATGCATGTCA |  |
| PIGA gRNA intr2_5 R | aaacTGACATGCATTACCTTCCAC |  |
| PIGA gRNA intr3_1 F | CACCGattgatataaatctaatGCA |  |
| PIGA gRNA intr3_1 R | aaacTGCattagatttatatcaatC |  |
| PIGA gRNA intr3_2 F | CACCGTGGTGATACTAATCTTGAGG |  |
| PIGA gRNA intr3_2 R | aaacCCTCAAGATTAGTATCACCAC |  |
| PIGA gRNA intr5_1 F | CACCGAATTgatggtgactccctag |  |
| PIGA gRNA intr5_1 R | aaacctagggagtcaccatcAATTC |  |
| PIGA gRNA Ex2_1 F | aaacCACCGTGCTCAGGTACATATTTGTT |  |
| PIGA gRNA Ex2_1 R | AACAAATATGTACCTGAGCA |  |
| qPCR PARP1 F | GATGGTGTAGACGTTCCTCTTG |  |
| qPCR PARP1 R | AATTCATACCAGAGCCACCG |  |
| qPCR LIG3 F | GGGAAGCCATCTAAGATCACG |  |
| qPCR LIG3 R | CACACAGAACCGTTGCTCAG |  |
| qPCR POLQ F | CTGCGTCGGAGTGGGAAAC |  |
| qPCR POLQ R | CTGTAGGCTTGCATTCTCCTG |  |
| qPCR RPA1F | CGGGAATGGGTTCTACTGTTTC |  |
| qPCR RPA1 R | CGAGCACAAATGGTCCACTTG |  |
| qPCR RPA2 F | GCACCTTCTCAAGCCGAAAAG |  |
| qPCR RPA2 R | CCCCACAATAGTGACCTGTGAAA |  |
| qPCR RPA3 F | AGCTCAATTCATCGACAAGCC |  |
| qPCR RPA3 R | TCTTCATCAAGGGGTTCCATCA |  |
| SH2B3 genotyping F | TCATGATCCTTCCGACAGAG |  |
| SH2B3 genotyping R | GAGAGCCGGACATCACA |  |
| PIGA-umi-F (10N, uf5) | ﻿CATCTTACGATTACGCCAACCACTGNNNNNTGNNNNNCGATCGTGGAAGCAGCCAGTTG |  |
| Universal F | CATCTTACGATTACGCCAACCACTG |  |
| PIGA -umi-R | ﻿TCCACTGCTTCCCACAAAAGGAGT |  |
| GFP repair ssODN | ﻿gatgcccttcagctcgatgcggttcaccagggtgtcgccctcgaacttcacctcggcgcgggtcttgtagttgccgtcgtccttgaagaagatggtgcgctcctggacgtagccttcggg |  |
| 5’Cy3-ssODN | /5Cy3/gatgcggttcaccagggtgtcgccctcgaacttcacctcggcgcgggtcttgtagttgccgtcgtccttgaagaagatggtgcgctcctg |  |
| GFPmut gRNA sequence | CAGGGTAATCTCGAGAGCTT |  |
| PIGA gRNA sequence in Fig. s1c | ATGTAGTAGCTGATGTATAG |  |
| PIGA gRNA intr1_1 sequence in Fig. s1d | TGGTAAACCATGATATGCTG | [6] |
| CD9 gRNA sequence in Fig. 1c | AACATCTGTGGACCCTGCAC |  |
| CD9 gRNA intr1 | GGGGTTAGGGCGACTAGGCG |  |
| CD9 gRNA intr2 | GGGGGTTCTGAATACGAGTG |  |
| CD9 gRNA intr5_1 | GCTGAACAAAGTGCCCAGCC |  |
| CD9 gRNA intr5_2 | AGAGCTTAGAGAGAACAAGA |  |
| CD9 gRNA intr5_3 | TCTTGAGACTAGTCAAGCG |  |
| CD9 gRNA intr6 | GACGATTAGAATGTTTGCAC |  |
| CD9 gRNA intr7 | AGAAAGAGATGGGTGCCCTG |  |
| LAMP2 gRNA intr2 | ACATACACGAATTTATCCAG |  |
| LAMP2 gRNA intr4 | GTGGGAGCAGATAAAGCATA |  |
| CD9 7kb F | GCCCTTCTCTCACCTCCTTT |  |
| CD9 7kb R | CTGGTGCTGACTCCTCTGTT |  |
| WAS 7kb F | CTCCCAAATCCAGACACCCT |  |
| WAS 7KB R | GCCAAGCCTTTTCTCACCTC |  |
| HBB 7kb F | TTTCCTGATTCTCCCACCCC |  |
| HBB 7kb R | AGGAAGGGAAGAGAGGACGT |  |
| EPOR ddPCR F | TCCTGCTCATCTGCTTTGG |  |
| EPOR ddPCR R | CATCTGCAGCCTGGTGTCC |  |
| EPOR G>A probe | TTGCGTCCATAGACACTGTGC (HEX ZEN / Iowa Black FQ) |  |
| EPOR ref probe1 | TGGCCCCTACTCCAACCCTTA (FAM ZEN / Iowa Black FQ) |  |
| EPOR 100bp ssDNA | AGCTTTGAGTACACTATCCTGGACCCCAGCTCCCAGCTCTTGCGTCCATAGACACTGTGCCCTGAGCTGCCCCCTACCCCACCCCACCTAAAGTACCTGT |  |
| WAS ddPCR F | GCTTGTCTCCTCGCCTTATT |  |
| WAS ddPCR R | GTATGGAAGCAGGGTCTCAC |  |
| WAS I294T probe | TCTACGACTTCACTGAGGACCAGG (HEX ZEN / Iowa Black FQ) |  |
| WAS ref probe | ATTCCTCTACTCCTGCCCCTGG (FAM ZEN / Iowa Black FQ) |  |
| WAS I294T ssDNA | CGAGGCCCAGCTCACCGACGCCGAGACCTCTAAACTTATCTACGACTTCACTGAGGACCAGGGTGGGCTGGAGGCTGTGCGGCAGGAGATGAGGCGCCAG |  |
| WAS I294T sgRNA | ACTTATCTACGACTTCATTG |  |
| TBP F | GATATGAGACTGTGGGTAAGT |  |
| TBPR | GATCCTTTGAACACCCTAATG |  |
| TBP_probe | ACAGAGATCACTGCAGTTGC (5' Hex/ZEN/3'IBFQ) |  |
| Xp22.31 VCX F | CCATGTAGGTCAGGCTGGTC | [35] |
| Xp22.31 VCX R | CGGAGGGCTATATGAAGACG | [35] |
| Xp22.31 PNPLA4 F | TGGATTTCTGGGCATTTACC | [35] |
| Xp22.31 PNPLA4 R | ACAGAAGCAACCAACGATCC | [35] |
| Xp11.4 TSPAN7 F | TTTCTCTCCCTTCCCCTACC | [35] |
| Xp11.4 TSPAN7 R | GTGGGGTCAGGAATAACTGG | [35] |
| Xp11.4 USP9X F | AGCGTGTCTGTGTGTTTTGG | [35] |
| Xp11.4 USP9X R | CGGAGACTCCATCCTCCTAC | [35] |
| Xp11.23 USP27X F | TCTACCAGTGCTTCGTGTGG | [35] |
| Xp11.23 USP27X R | CAGGAAAGGCAAGAGTGGAG | [35] |
| Xq26.2 HPRT1 F | CCTGGGAAAAGAGGACTGC | [35] |
| Xq26.2 HPRT1 R | CATCATTCCCGAATCTGC | [35] |
