# Supplementary figures and images for: Modulation of the microhomology-mediated end joining pathway suppresses large deletions and enhances homology-directed repair following CRISPR-Cas9-induced DNA breaks
Source: BMC Biol. 2024 Apr 29;22:101. doi: 10.1186/s12915-024-01896-z (PMC11059712; doi:10.1186/s12915-024-01896-z)

Figure S2b

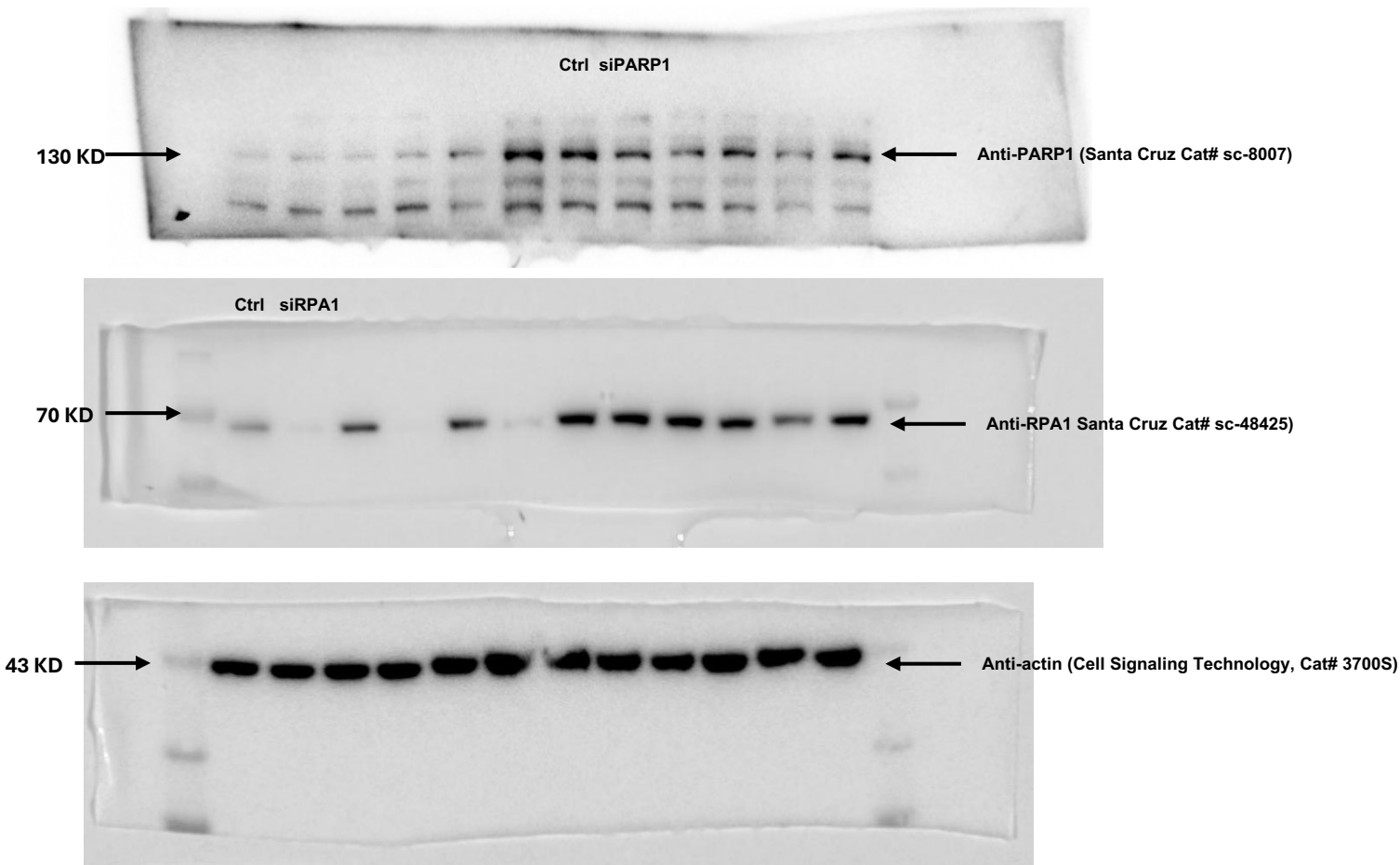

Figure S4a

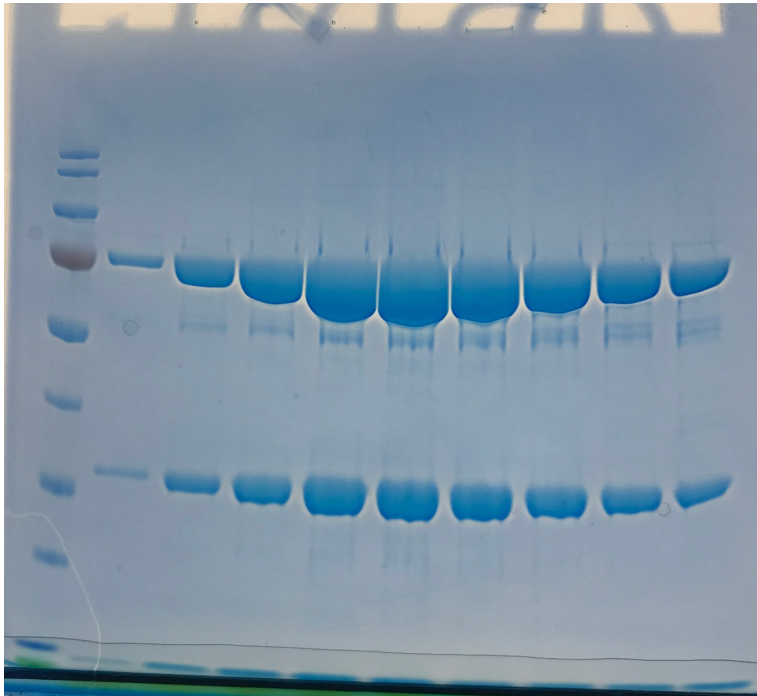

Supplement: Supplementary file 3 — Additional file 3: Original blots and gels. [file 12915_2024_1896_MOESM3_ESM.pdf]
